# Examining modification of the associations between air pollution and birth outcomes by neighborhood deprivation in a North Carolina birth cohort, 2011–2015

**DOI:** 10.3389/frph.2024.1304749

**Published:** 2024-07-11

**Authors:** Kristen N. Cowan, Alison K. Krajewski, Monica P. Jimenez, Thomas J. Luben, Lynne C. Messer, Kristen M. Rappazzo

**Affiliations:** ^1^ Oak Ridge Institute for Science and Education (ORISE), US EPA, Research Triangle Park, NC, United States; ^2^ Department of Epidemiology, Gillings School of Global Public Health, University of North Carolina at Chapel Hill, Chapel Hill, NC, United States; ^3^ Office of Research and Development, Center for Public Health & Environmental Assessment, United States Environmental Protection Agency, Research Triangle Park, NC, United States; ^4^ Departments of Community Health and Health Promotion and Epidemiology, OHSU-PSU School of Public Health, Portland, OR, United States

**Keywords:** air pollution, birth outcomes, neighborhood deprivation, modification, preterm birth, birth defects

## Abstract

**Background:**

Evidence from studies of air pollutants and birth outcomes suggests an association, but uncertainties around geographical variability and modifying factors still remain. As neighborhood-level social characteristics are associated with birth outcomes, we assess whether neighborhood deprivation level is an effect measure modifier on the association between air pollution and birth outcomes in a North Carolina birth cohort.

**Methods:**

Using birth certificate data, all North Carolina residential singleton live births from 1 January 2011 to 31 December 2015 with gestational ages of 20–44 weeks (*n* = 566,799) were examined for birth defect diagnoses and preterm birth. Exposures were daily average fine particulate matter (PM_2.5_), daily 8-h maximum nitrogen dioxide (NO_2_), and daily 8-h maximum ozone (O_3_) modeled concentrations, and the modifier of interest was the neighborhood deprivation index (NDI). Linear binomial models were used to estimate the prevalence differences and 95% confidence intervals (CI) for the association between ambient air pollution and birth defect diagnoses. Modified Poisson regression models were used to estimate risk differences (RDs) and 95% CIs for air pollution and preterm birth. Models were stratified by the neighborhood deprivation index group (low, medium, or high) to assess potential modification by NDI.

**Results:**

Approximately 3.1% of the study population had at least one birth defect and 8.18% were born preterm. For preterm birth, associations with PM_2.5_ and O_3_ did not follow a conclusive pattern and there was no evidence of modification by NDI. The associations between NO_2_ and preterm birth were generally negative across exposure windows except for a positive association with NO_2_ and preterm birth for high NDI [RD: 34.70 (95% CI 4.84–64.56)] for entire pregnancy exposure. There was no evidence of associations between pollutants examined and birth defects.

**Conclusions:**

There may be differences in the association between NO_2_ exposure and preterm birth by NDI but we did not observe any evidence of associations for birth defects. Our results support the public health protection afforded by reductions in air pollution, even in areas of neighborhood deprivation, but future research conducted in areas with higher levels of air pollution and evaluating the potential for modification by neighborhood deprivation level would be informative.

## Introduction

Preterm birth (PTB) and birth defects are two of the leading causes of infant mortality in the United States (1–3). These adverse birth outcomes are also associated with high medical expenses, higher risk of medical conditions across the life course, and developmental disabilities (4–9). Across live born infants in the United States since 2010, about 10% (9.57–10.49) were preterm, defined as being born before 37 weeks of gestation (10); and about 3% had at least one birth defect diagnosed in their first year of life. Birth defects can range in severity with over 1,000 different types identified (11). There are several established risk factors for adverse birth outcomes, e.g., age of the birthing person and smoking for preterm birth (12–14), and heavy alcohol use, certain medications, and uncontrolled diabetes for birth defects (15–19). Researchers have also reported associations between environmental exposures, such as lack of greenspace, extreme heat, and air pollution, and adverse birth outcomes (20–31).

Over the past few decades, evidence has accumulated suggesting an association between air pollution and birth outcomes. A number of studies have identified a positive association between increases in exposure to air pollutants (i.e., NO_2_, PM_2.5_, O_3_) and preterm birth (32–34), with some studies finding that exposure to O_3_ specifically during the first two trimesters of pregnancy heightens the risk of preterm birth (32). While most studies have found positive associations between air pollution and preterm birth, a 2020 systematic review identified five (among 24 total studies on PM_2.5_ and preterm birth) that did not find an association when measuring exposure across pregnancy or by trimester (35). However, evidence regarding the association between air pollution and birth defects is less consistent. Some studies have reported associations between NO_2_ and congenital heart defects (36), but others have not (37), and findings between O_3_ and PM_2.5_ with birth defects are very inconsistent (32, 34). Given the heterogeneity reported across studies, there are uncertainties around the role of exposure timing, birthing parent characteristics, geographical variability, and other mediating or modifying factors in the relationship between air pollution and poor birth outcomes (38). In addition, there is limited knowledge on the mechanisms by which air pollution may affect birth outcomes (39, 40).

Given the well-documented sociodemographic disparities in both air pollution exposure, particularly with higher levels of pollution occurring in areas with a higher proportion of non-White residents (41–43), and racial disparities observed in birth defects and preterm birth (44–47), structural factors may potentially modify air pollutant–birth outcome associations. Research has also shown that neighborhood-level characteristics such as neighborhood deprivation are associated with poor birth outcomes (48–53). Environmental and social factors likely contribute to cumulatively impact birth outcomes, worsening disparities (54, 55). However, there is limited information on how air pollutants and neighborhood deprivation interact with one another to impact birth outcomes.

To address this gap, the goal of this study was to examine if the neighborhood deprivation level is an effect measure modifier on the association between air pollution and birth outcomes using data from all eligible births in North Carolina (NC).

## Materials and methods

### Study population

Birth certificate data for live births were provided by the NC Department of Health and Human Services’ Division of Public Health linked with data from the Birth Defects Monitoring Program (*n* = 588,135). The population for this study included all live, singleton births with gestational age between 20 and 44 weeks delivered from 1 January 2011 to 31 December 2015 with residence at delivery in NC. These specific years were selected due to data availability and consistency of records as a result of changes made to the North Carolina birth cohort in 2010. After excluding multiple births (*n* = 20,586), deliveries outside of 20–44 weeks gestational age (*n* = 642), and missing residential address at time of delivery that precluded geocoding (*n* = 8), the final sample was 566,799 births. The residential address at time of delivery was geocoded to the corresponding census tract using ArcGIS (ESRI, version 10.8, Redlands, CA).

### Birth outcomes

The outcomes of interest in this study were birth defects and preterm birth. Birth defects were identified from the North Carolina birth defect monitoring program (NCBDMP), which is a surveillance system that uses medical records to identify all birth defects by type that are diagnosed in the first year of life in NC (56). We chose birth defects with previous evidence of associations with air pollutant exposures to examine in this analysis: these included pulmonary valve atresia/stenosis, tetralogy of Fallot, atrioventricular septal defects, and lower limb reduction defects; we also examined gastroschisis due to higher prevalence among non-White births (57–59).

PTB was defined as delivery at less than 37 weeks completed gestation based on clinical estimate of gestational age as reported on the birth certificate. All live, singleton births without any birth defects, gestational age between 20 and 44 weeks, and birth weight between 1,000 and 6,000 g were included in analyses of preterm birth.

### Air pollution exposures

The air pollutants of interest in this study were daily average fine particulate matter (PM_2.5_), daily 8-h maximum nitrogen dioxide (NO_2_), and daily 8-h maximum ozone (O_3_) concentrations. Daily census tract–level concentration estimates for daily average PM_2.5_ and 8-h maximum for O_3_ came from the EPA's Fused Community Multiscale Air Quality model surface using Downscaling (fCMAQ) model. The fCMAQ model links observed pollution data from EPA monitoring sites with deterministic chemistry and meteorology data from the Community Multiscale Air Quality model through a spatially and temporally varying coefficient model (60–62). These data are available for download at RSIG-related downloadable data files (https://www.epa.gov/hesc/rsig-related-downloadable-data-files). Daily 8-h maximum NO_2_ concentration estimates were extracted from a hybrid ensemble model with 1 km^2^ spatial resolution, which used multiple machine learning algorithms and predictor variables, including satellite data, meteorological variables, land-use variables, elevation, and chemical transport model predictions to estimate daily concentrations for NO_2_ at 1 km^2^ grid resolution (63). PM_2.5_ and O_3_ data were provided at the census tract level, and NO_2_ data were aggregated to census tract level. Births were then linked to air pollutant concentrations by census tract of residential address at time of birth, and daily concentrations of PM_2.5_, NO_2_, and O_3_ were averaged across each trimester. Trimester 1 was considered to go through week 12, and trimester 2 began at the start of week 13 and continued through week 26.

### Modifier

A neighborhood deprivation index (NDI) was created using principal component analysis on 2010 census variables including housing, poverty, employment, occupation, and education at the census tract level (64). The NDI used here is a relative ranking of neighborhood deprivation for NC census tracts compared to one another; a higher index value is interpreted as having more deprivation. Neighborhood deprivation categories were determined by visually examining the distribution of NDI across all census tracts in NC and using a nearest centroid sorting clustering method to group census tracts into three NDI levels (low, medium, or high deprivation) (65). Individuals were assigned an NDI category based on census tract of the residential address at time of birth.

### Covariates

Covariates of interest in this study were obtained from the birth certificate records and included birthing parent demographic characteristics of age at delivery, race/ethnicity (white, non-Hispanic, Black, non-Hispanic; Hispanic; Asian or Pacific Islander, non-Hispanic; American Indian, non-Hispanic; other), marital status (married or unmarried), Medicaid status at time of delivery (yes or no), education (<high school, high school, >high school), and month of conception (estimated using clinical estimate of gestational age and birth date). In the context of these analyses, race and ethnicity are used to represent potential stress from experiencing interpersonal and structural racism as well as residential segregation within the US and not as a biological construct (66).

### Statistical analysis

As the objective of these analyses was to evaluate the potential for effect measure modification of air pollutant–birth outcome associations by neighborhood deprivation, unstratified and NDI-stratified (low, medium, and high) models were run. The presence of effect measure modification was evaluated qualitatively by examining the separation of stratified effect estimates from the unstratified effect estimate. Confounders of interest were determined using a directed acyclic graph (DAG, Supplementary Figure S1) (67).

Linear binomial regression models were used to estimate the prevalence differences (PDs) and 95% confidence intervals (CIs) for the association between ambient air pollution concentration and individual birth defect diagnoses for any birth defects and specific birth defect types. Any birth defect includes all diagnosed birth defect phenotypes and not just the specific phenotypes examined individually. The associations were estimated per 10,000 births for each individual air pollutant (PM_2.5_, NO_2_, and O_3_). Exposure contrasts for each pollutant were approximately 10% increases with values: 1 µg/m^3^ increase for PM_2.5_, 4 ppb increase in O_3,_ and 7 ppb increase in NO_2_. Exposures were assigned as the average daily pollutant concentration across the first trimester (weeks 1–12). Models for birth defects were adjusted for birthing parent age at delivery (centered at age 26 with a quadratic term), race/ethnicity (white, non-Hispanic as reference), and education (>high school as reference).

Modified Poisson regression models were used to estimate risk differences (RDs) and 95% CIs for air pollution and PTB. The associations were estimated per 10,000 births for each individual air pollutant (PM_2.5_, NO_2_, and O_3_), with approximately 10% increases as reported above. Exposures were assigned as the average daily pollutant concentration per each day, each gestational week, first and second trimester, and the entire pregnancy. We did not examine third trimester due to inconsistent exposure windows for PTBs. Models for PTB were adjusted for birthing parent race/ethnicity (white, non-Hispanic as reference), birthing parent age at delivery (centered at age 26 with a quadratic term), marital status (married as reference), Medicaid status (no as reference), education (>HS as reference), and month of conception (index variable as referent).

Linear binomial regression models were fit for birth defects rather than modified Poisson regression models due to low count numbers of specific defects but were checked across model types where possible and produced identical results.

Statistical analyses were performed using SAS (version 9.4; Cary, NC). All figures were created using R (version 4.1.0; Vienna, Austria; packages: ggplot2).

### Sensitivity analyses

Due to the relatively low prevalence of birth defects in comparison to PTB, we were unable to adjust for the same number of covariates across models for birth defects and PTB. We conducted sensitivity analyses using linear binomial regression to estimate the PDs for the association between air pollution and birth defects stratified by NDI adjusting for marital status and month of conception in addition to the current adjustment set reported. Due to small cell counts, the convergence of these models was uncertain. For specific birth defects, (pulmonary valve atresia/stenosis, tetralogy of Fallot, atrioventricular septal defects, and lower limb reduction defects), sensitivity analyses were conducted using the exposure over weeks 3–8 only due to previous literature on sensitive windows for these outcomes (28, 36, 68). In addition, sensitivity analyses were conducted examining the interaction between NDI and air pollution, with both terms and an interaction term in the linear models. For interaction effects, we set an alpha level of 0.10.

### IRB approval/human subjects research approval

This analysis was approved as minimal risk/existing data under the University of North Carolina (IRB) (09–0828). This study was also approved as observational research involving human subjects by the EPA's Human Subject's Research Review Official [HSRRO Project # F09-019CS].

## Results

### Descriptive information on this cohort and outcomes

Overall, there were 566,799 live births eligible for our study of birth defects from 2011 to 2015. Among those, 17,691 (3.1%) had at least one birth defect, 479 (0.08%) had pulmonary valve atresia or stenosis, 245 (0.04%) had tetralogy of Fallot, 299 (0.05%) had atrioventricular septal defects, and 80 (0.01%) had a lower limb reduction defect. Among the 566,512 birthing parent–infant pairs included in the analysis on PTB (which excluded those with any birth defects), there were 46,289 (8.17%) PTB cases. The majority of the full population (55.81%) identified as white, non-Hispanic, 23.67% identified as Black, non-Hispanic, 15.04% as Hispanic, 3.92% as Asian/Pacific Islander, non-Hispanic, 1.31% as American Indian, non-Hispanic, and 0.25% as another race. Among this population, the majority (60.0%) had more than a high school diploma, 22.50% completed high school, and another 17.19% completed less than high school. About 55.38% of the people who gave birth in this population were on Medicaid at the time of birth and 41.03% were unmarried. Demographic characteristics by outcome are presented in Table 1.

**Table 1:** Descriptive information on cohort of births from 2011 to 2015 (*N* = 566,799).

Birthing person information	Total population	Among those with preterm birth	Among those with any birth defect
	*N* (%)	*N* (%)	*N* (%)
Race/ethnicity			
White, non-Hispanic	316,331 (55.81)	21,091 (94.70)	10,395 (58.76)
Black, non-Hispanic	134,148 (23.67)	13,384 (31.54)	4,004 (22.63)
Hispanic	85,262 (3.92)	5,798 (13.66)	2,510 (14.19)
Asian/Pacific Islander, non-Hispanic	22,239 (3.92)	1,411 (3.32)	478 (2.70)
American Indian, non-Hispanic	7,408 (1.31)	634 (1.49)	270 (1.53)
Other, non-Hispanic/unknown	1,411 (0.25)	120 (0.28)	34 (0.19)
Education level			
Less than high school	97,445 (17.19)	96,681 (17.18)	3,190 (18.07)
Completed high school	127,506 (22.50)	126,324 (22.45)	4,188 (23.73)
More than high school	340,073 (60.00)	337,861 (60.05)	10,274 (3.02)
Medicaid status	313,903 (55.38)	26,475 (62.39)	10,618 (60.02)
Unmarried	232,555 (41.03)	20,663 (48.69)	7,646 (43.23)
Outcomes of interest			
Preterm birth	42,438 (7.54)	—	—
Any Birth defect	17,691 (3.12)	—	—
Gastroschisis	213 (0.04)		
Pulmonary valve atresia/stenosis	479 (0.08)	—	—
Tetralogy of fallout	245 (0.04)	—	—
Atrioventricular septal defects	299 (0.05)	—	—
Limb reduction defects	80 (0.01)	—	—

### Exposure and modifier information

Across North Carolina from 2011 to 2015, average daily PM_2.5_ exposure during trimester 1 ranged from 5.05 to 22.36 µg/m^3^ with an interquartile range (IQR) of 8.55–10.47 µg/m^3^. The average daily O_3_ exposure during trimester 1 ranged from 26.93 to 60.54 ppb with an IQR of 34.73–46.15 ppb. The daily average NO_2_ exposure across trimester 1 ranged from 0.32 to 41.73 ppb with an IQR of 9.63–17.53 ppb. The exposures did not differ substantially across trimester or neighborhood deprivation level (Tables 2,3). Neighborhood deprivation levels across the state ranged from −2.00 to 4.39 (Supplementary Figure S2) and the IQR for NDI was −0.63 to 0.65. The low NDI group ranged from −2.000 to −0.323, the medium NDI group ranged from −0.324 to 0.986, and the highest NDI group ranged from 0.987 to 4.390. The magnitude of pollutant exposure did not differ across NDI levels (Tables 2,3).

**Table 2:** Descriptive statistics for exposure to PM2.5 (μg/m_3_), O_3_ (ppb), NO_2_ (ppb) among preterm births stratified by NDI level.

	Min	25th pctl	Median	75th pctl	Max
Overall					
PM_2.5_ (µg/m^3^) during trimester 1	5.05	8.55	9.41	10.47	22.36
O_3_ (ppb) during trimester 1	26.93	34.73	40.31	46.15	60.54
NO_2_ (ppb) during trimester 1	0.32	9.63	13.28	17.53	41.73
NDI at birth	−2.00	−0.63	−0.02	0.65	4.39
Among low NDI cluster					
PM_2.5_ (µg/m^3^) during trimester 1	5.47	8.67	9.52	10.62	21.09
O_3_ (ppb) during trimester 1	27.04	34.44	40.19	46.18	60.46
NO_2_ (ppb) during trimester 1	0.32	10.61	14.26	18.28	41.73
NDI at birth	−2.00	−1.21	−0.84	−0.58	−0.32
Among medium NDI cluster					
PM_2.5_ (µg/m^3^) during trimester 1	5.05	8.42	9.26	10.31	22.36
O_3_ (ppb) during trimester 1	26.93	35.06	40.44	46.11	60.36
NO_2_ (ppb) during trimester 1	0.33	8.64	11.79	15.85	36.61
NDI at birth	−0.32	−0.07	0.20	0.53	0.99
Among high NDI cluster					
PM_2.5_ (µg/m^3^) during trimester 1	5.85	7.97	9.53	10.52	18.27
O_3_ (ppb) during trimester 1	27.00	34.37	40.29	46.23	60.54
NO_2_ (ppb) during trimester 1	1.11	11.15	15.52	19.74	34.82
NDI at birth	0.99	1.17	1.46	1.95	4.39

min, minimum; 25th pctl, 25th percentile; 75th pctl, 75th percentile; max, maximum.

**Table 3:** Descriptive statistics for exposure to PM2.5 (μg/m_3_), O_3_ (ppb), NO_2_ (ppb) among preterm births stratified by NDI level.

Exposure or modifier	Min	25th pctl	Median	75th pctl	Max
Overall					
NDI	−2.00	−0.48	0.11	0.82	4.39
PM_2.5_ (µg/m^3^)					
Entire pregnancy	6.03	8.73	9.52	10.24	14.93
Trimester 1	5.07	8.54	9.39	10.43	19.83
Trimester 2	5.23	8.51	9.32	10.32	21.79
O_3_ (ppb)					
Entire pregnancy	30.34	37.80	40.33	43.03	55.14
Trimester 1	27.34	34.79	40.45	46.08	60.23
Trimester 2	26.13	35.11	40.59	45.73	60.53
NO_2_ (ppb)					
Entire pregnancy	1.00	9.84	13.14	17.24	33.42
Trimester 1	0.36	9.53	13.17	17.42	37.08
Trimester 2	0.78	9.44	12.92	17.24	39.89
Among low NDI cluster					
NDI	−2.00	−1.15	−0.80	−0.55	−0.32
PM_2.5_ (µg/m^3^)					
Entire pregnancy	6.17	8.85	9.69	10.38	13.21
Trimester 1	5.51	8.65	9.53	10.58	19.83
Trimester 2	5.23	8.61	9.44	10.48	20.11
O_3_ (ppb)					
Entire pregnancy	30.34	37.67	40.27	43.06	55.14
Trimester 1	27.34	34.61	40.45	46.23	60.23
Trimester 2	26.13	34.86	40.54	45.90	60.53
NO_2_ (ppb)					
Entire pregnancy	2.24	10.72	14.03	17.54	30.80
Trimester 1	0.42	10.39	14.01	17.99	36.27
Trimester 2	1.73	10.16	13.78	17.87	39.89
Among medium NDI cluster					
NDI	−0.32	−0.06	0.22	0.55	0.99
PM_2.5_ (µg/m^3^)					
Entire pregnancy	6.03	8.59	9.35	10.10	13.58
Trimester 1	5.07	8.42	9.25	10.27	19.39
Trimester 2	5.57	8.37	9.18	10.16	20.10
O_3_ (ppb)					
Entire pregnancy	30.38	37.95	40.43	43.00	53.79
Trimester 1	27.42	35.04	40.50	45.95	60.23
Trimester 2	26.22	35.42	40.68	45.59	60.32
NO_2_ (ppb)					
Entire pregnancy	1.00	9.00	11.69	15.42	32.50
Trimester 1	0.36	8.56	11.69	15.76	37.08
Trimester 2	0.78	8.57	11.55	15.54	37.37
Among high NDI cluster					
NDI	0.99	1.18	1.52	1.98	4.39
PM_2.5_ (µg/m^3^)					
Entire pregnancy	6.74	8.93	9.63	10.32	14.93
Trimester 1	6.09	8.67	9.53	10.50	17.65
Trimester 2	6.15	8.63	9.47	10.41	21.79
O_3_ (ppb)					
Entire pregnancy	30.82	37.68	40.19	43.06	53.37
Trimester 1	27.40	34.47	40.33	46.10	59.90
Trimester 2	27.10	34.71	40.45	45.82	60.45
NO_2_ (ppb)					
Entire pregnancy	1.69	11.75	15.89	19.15	33.42
Trimester 1	1.75	11.30	15.69	19.91	34.72
Trimester 2	1.64	11.08	15.25	19.59	33.86

min, minimum; 25th pctl, 25th percentile; 75th pctl, 75th percentile; max, maximum.

### Risk differences for preterm birth

**Figure 1:**
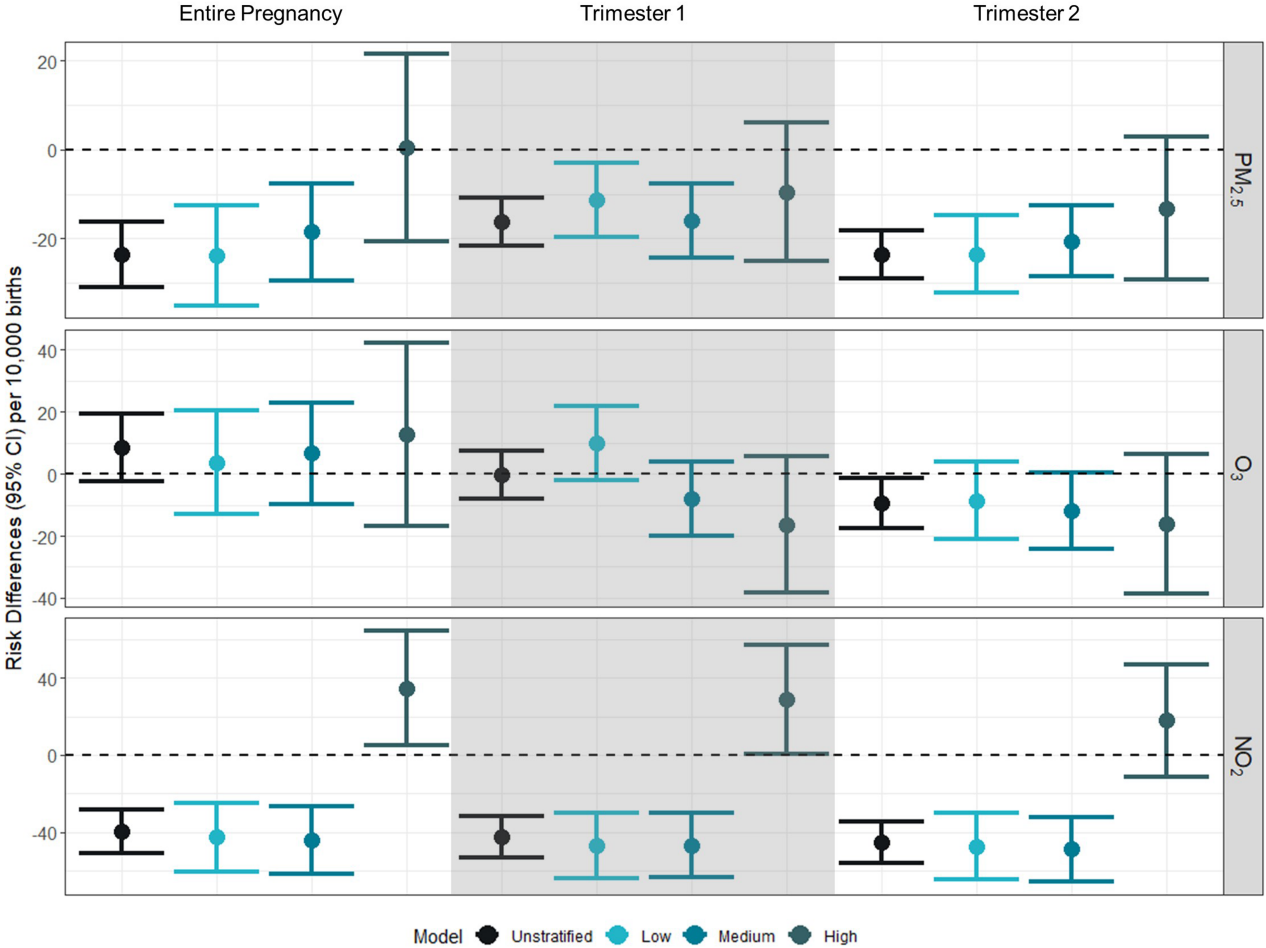
Risk differences (95% CI) for gestational exposure to PM_2.5_, O_3_, and NO_2_ and preterm birth per 10,000 births. Risk differences represent the absolute increase in number of preterm births per 10,000 associated with a 10% increase in the exposure of interest (PM_2.5_: 1 µg/m^3^; O_3_: 4 ppb; NO_2_: 7 ppb). Unstratified models were adjusted for birthing parent race/ethnicity (white, non-Hispanic as reference), birthing parent age at delivery (centered at age 26 with a quadratic term), marital status (married as reference), Medicaid status (no as reference), education (>HS as reference), and month of conception (index variable as referent). Models were further stratified by NDI levels at low (−2 to −0.323), medium (−0.324 to 0.986), high (0.987–4.39).

Associations between PM_2.5_ and PTB were generally negative among low and medium NDI strata across the entire pregnancy, but there were positive leaning associations for those residing in a high NDI; however, these generally had confidence intervals that overlapped with the null value of 1 (Figure 1 and Supplementary Table S1). Despite this, for the associations between PM_2.5_ and PTB, there was no statistical evidence of modification by NDI with confidence intervals overlapping.

Across time periods and NDI values, the relationship was generally insignificantly different from the null value of 1, across exposure windows. There was a negative association [RD: −9.32 (95% CI: −17.44 to −1.19)] between O_3_ and PTB in the second trimester in the adjusted model. There was no evidence of modification by NDI.

We observed negative associations between NO_2_ and preterm birth for the entire pregnancy for the adjusted model [RD: −39.53 (95% CI: −50.77 to −28.28)], low NDI [RD: −42.78 (95% CI: −60.80 to −24.76)], and medium NDI [RD: −44.24 (95% CI: −61.79 to −26.68)], but a positive association was observed between NO_2_ and preterm birth for the entire pregnancy among those residing in a high NDI [RD: 34.70 (95% CI: 4.84–64.56)]. This pattern of association was similar for trimesters 1 and 2, with negative associations between NO_2_ and preterm birth for the adjusted models, low NDI, and medium NDI, and a positive association between NO_2_ and preterm birth for high NDI. When comparing across strata of the NDI, we see evidence of effect modification for NO_2_ by NDI level across all exposure windows.

### Prevalence differences for birth defects

**Figure 2:**
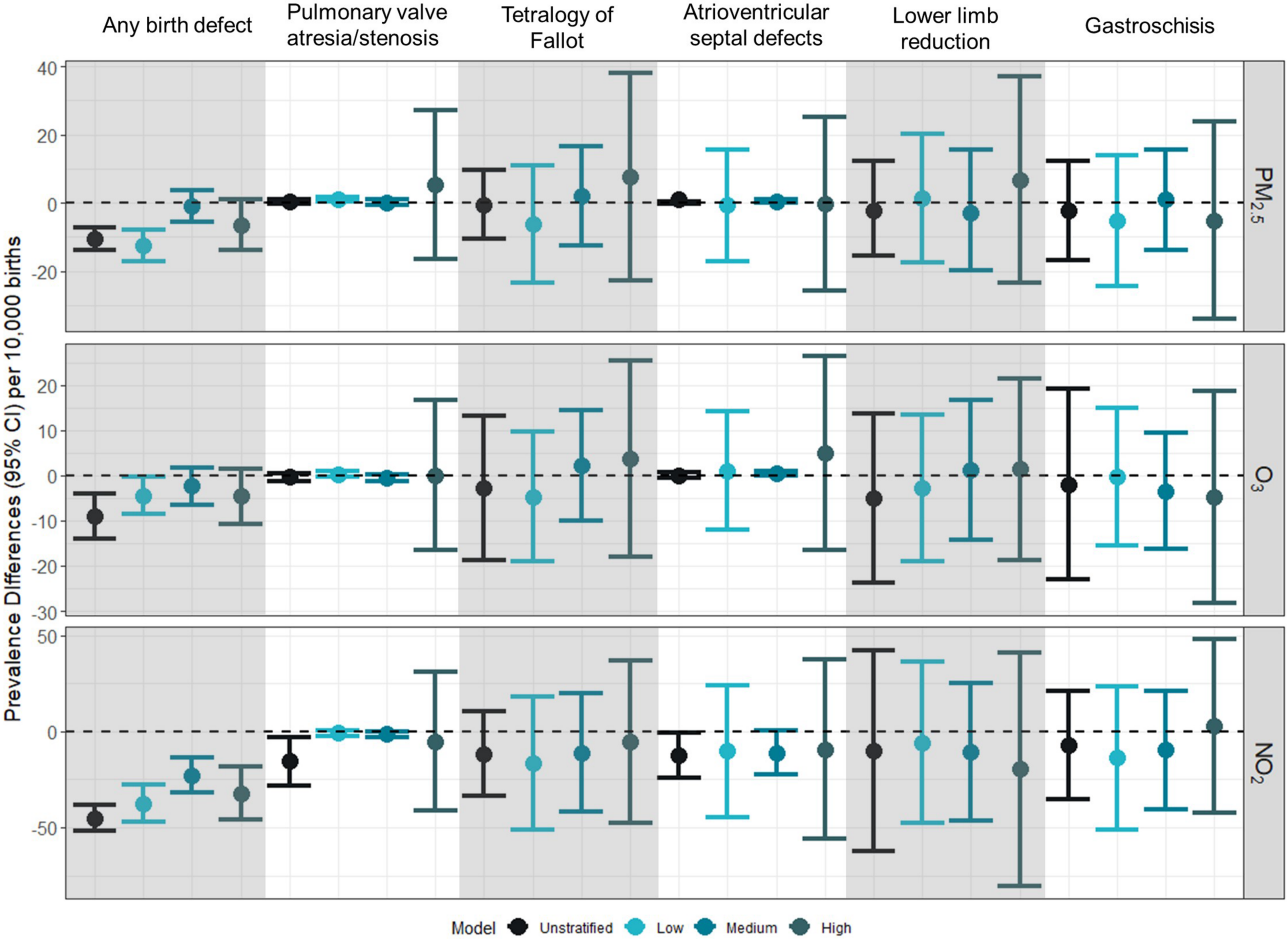
Prevalence difference (95% CI) for first trimester exposure to PM_2.5_, O_3_, and NO_2_ and selected birth defects per 10,000 births. Prevalence differences represent the absolute increase in the number of preterm births per 10,000 associated with a 10% increase in the exposure of interest (PM_2.5_: 1 µg/m^3^; O_3_: 4 ppb; NO_2_: 7 ppb). Unstratified models were adjusted for birthing parent age at delivery (centered at age 26 with a quadratic term), race/ethnicity (white, non-Hispanic as reference), and education (>HS as reference). Models were further stratified by NDI levels at low (−2 to −0.323), medium (−0.324 to 0.986), high (0.987–4.39).

In general, across all pollutants examined, no evidence of association was observed between pollutants and birth defect prevalence, and associations did not differ across NDI levels or trimesters (Figure 2 and Supplementary Table S2).

In the sensitivity analysis that additionally adjusted for month of conception and marital status, we observed associations that were not significantly different from the null value of 1 (Supplementary Table S3). When examining exposures across gestational weeks 3–8, only similar null associations were observed (Supplementary Table S4).

Results for sensitivity analyses examining the interaction between NDI and air pollution for PTB and birth defects are shown in Supplementary Tables S5 and S6, respectively.

## Discussion

### Summary of results

Overall, we did not observe any strong associations between PM_2.5_, O_3_ and NO_2_ with prevalence of birth defects in this cohort of singleton live births in North Carolina from 2011 to 2015 and stratifying by neighborhood deprivation level did not substantially alter these associations. In terms of preterm birth, associations with PM_2.5_ were generally negative. It is possible that areas with higher levels of PM_2.5_ are also areas with higher access to resources and high-quality healthcare leading to negative associations between PM_2.5_ and poor birth outcomes. Modest associations were observed between O_3_ exposure during trimester 3 and preterm birth for those in neighborhoods considered to be at medium and high levels of neighborhood deprivation. In earlier trimesters, negative associations were generally observed between O_3_ exposure and preterm birth. Across all trimesters, there was an increased risk of preterm birth associated with NO_2_ exposure for those in highly deprived neighborhoods.

We evaluated air pollution exposures over trimesters of pregnancy. Previous studies have examined weeks and months of pregnancy as well (69, 70), though inconsistency in the exposure window associated with preterm birth is a noted uncertainty (32–34). For example, Krajewski et al. (70) reported an increased risk of PTB associated with PM_2.5_ and O_3_ exposure across gestational weeks and generally null effects for NO_2_. Alman et al. (69) observed elevated odds of preterm birth with PM2.5 exposure in months 3 and 4 as well as weeks 9–12, while Wang et al. reported increased hazard ratios for weeks 20–28 for PM_2.5_, 18–31 for NO_2_, and 23–31 for O_3_ (71). In general, the results of weekly, monthly, and trimester-specific average air pollutant exposure concentrations have yielded inconsistent associations with preterm birth, and no single exposure window has been identified as etiologically relevant.

We expected to see higher prevalence differences or risk differences for air pollution and birth outcomes for more deprived neighborhoods, but we only saw this for the association between NO_2_ and preterm birth. Since the inception of the Clean Air Act and the National Ambient Air Quality Standards in the United States, criteria air pollutant levels have steadily decreased over time (72, 73). It is possible that the levels for the other pollutants in North Carolina from 2011 to 2015 do not vary enough across the state to observe an association or that they are generally lower due to air pollution controls that have been put in place and have reduced air pollution. It is also possible that other correlated exposures that may exist in high deprivation neighborhoods are interacting with air pollution to create a higher association between NO_2_ and preterm birth such as noise pollution or limited green space. These other harmful exposures may be correlated with neighborhood deprivation due to environmental injustices in lower income neighborhoods.

### Context with other literature

There are few studies examining if neighborhood deprivation level modifies the association between air pollution and birth outcomes. In general, some studies have identified links between heightened exposure to air pollution and some specific birth defects and preterm birth during critical periods of pregnancy (59, 74–76); however, these studies are in places with higher levels of ambient air pollution than observed in North Carolina for this study period. Our results are consistent with another study that reported mostly null non-significant associations between air pollution and birth defects in North Carolina. A study similar to ours in New York City reported inverse associations between NO_2_ and birthweight in the most and least deprived neighborhoods indicating that the associations between NO_2_, neighborhood deprivation, and birth outcomes may be complicated (77). In addition, only a few studies have observed associations between neighborhood deprivation and the prevalence of birth defects overall, but one showed an association between increased neighborhood deprivation and higher prevalence of gastroschisis (50, 78). Some studies report positive associations with air pollution and preterm birth, especially with NO_2_ (79–81). Further research has identified that neighborhood deprivation is associated with increased preterm birth risk and one study demonstrated that neighborhood deprivation and urbanicity are associated with a higher risk, implying that there may be risks associated with the interaction between neighborhood deprivation and traffic-related air pollution (38, 64).

### Potential strengths and limitations

There are some limitations that may affect these results and their generalizability. Air pollutant exposures and neighborhood deprivation were assigned to parent–infant dyads based on the census tract they resided in at the time of birth, which does not account for any movement during pregnancy prior to birth or general day-to-day movement in areas outside of the census tract in which individuals live. Movement may be differential by neighborhood deprivation if people residing in more deprived areas need to leave their neighborhoods more often to access resources during pregnancy. A limitation specific to our birth defects analysis is that we were limited in how many covariates we could adjust for and in our ability to examine interaction between co-exposure to high levels of NDI and air pollution due to small counts of more rare birth defects during the study period. In addition, owing to the use of birth certificate records, we do not have access to behavioral factors or conditions that might exacerbate risk of adverse outcomes. In addition, by using birth certificate records, we had to use proxy measures for some covariates, such as partner status is defined using marital status in the birth certificate data and people who are unmarried and live with their partner may be classified as unmarried. We used spatiotemporal models to predict air pollution concentrations averaged at the census tract level. While these models provide well-validated predictions for PM_2.5_, O_3_, and NO_2_, they are not available for other criteria or hazardous air pollutants. In addition, we used modeled output at the census tract level for each of the three criteria pollutants examined even though there is known variability in the spatial heterogeneity of these three criteria pollutants.

Despite these limitations, there are several strengths of this study. By using this large North Carolina birth cohort, which includes all live births and registered birth defects identified through active case ascertainment, we were able to examine the associations for very rare birth defects. The use of modeled air pollution data allowed us to include all births for which the residence at delivery could be geocoded and linked with a census tract, including both urban and rural census tracts, regardless of the distance from a stationary monitor. The modeled air pollution data allowed us to predict daily concentrations and incorporated atmospheric conditions as part of the concentrations estimates.

In addition, we were able to examine possible critical periods for preterm birth by assigning exposures across trimesters. Previous work examined weekly exposure averages to the same pollutants but did not identify a consistent week or weeks of exposure thought to be critical for preterm birth. Thus, we chose to evaluate trimesters of exposure to facilitate comparison with results of other studies of air pollution or NDI and birth outcomes. The etiology for, and timing of, insults for the different birth defect phenotypes examined in our analyses varies. The critical window of exposure for congenital heart defects and limb defects includes gestational weeks 2–8, while the critical window of exposure for gastroschisis is later, gestational weeks 7–12. For ease of interpretation and to facilitate comparison of results with our analyses as well as with other published results, we used the first trimester as the exposure window of interest for all birth defect phenotypes.

### Public health implications

Overall, our study found a notable association between NO_2_ exposure and preterm birth, but we did not observe any strong associations for birth defects. This study examined modification by NDI and not the joint effect of neighborhood deprivation and air pollution, so further research measuring the joint effect of the two coexisting exposures would address this gap in data. In addition, this study was done in North Carolina where air pollution concentrations are relatively low. In general, our results support the public health protection afforded by EPA's National Ambient Air Quality Standards, even in areas of neighborhood deprivation. Future research conducted in areas with higher air pollution levels and evaluating the potential for modification by neighborhood deprivation level will be informative.

## Data Availability

The data analyzed in this study are subject to the following licenses/restrictions: data used in this study contain personally identifiable information and are not available without authorization from the NC Department of Health and Human Services. Requests to access these datasets should be directed to https://schs.dph.ncdhhs.gov/contacts.htm.

## Author disclaimer

The views expressed in this article are those of the author(s) and do not necessarily represent the views or policies of the U.S. Environmental Protection Agency. Mention of trade names or commercial products does not constitute endorsement or recommendation for use. This research was supported, in part, by appointments to the Research Participation Program for the U.S. Environmental Protection Agency, Office of Research and Development, administered by the Oak Ridge Institute for Science and Education (ORISE) through an interagency agreement between the U.S. Department of Energy and Environmental Protection Agency.
