# Supplementary material for: Examining modification of the associations between air pollution and birth outcomes by neighborhood deprivation in a North Carolina birth cohort, 2011–2015
Source: Front Reprod Health. 2024 Jul 11;6:1304749. doi: 10.3389/frph.2024.1304749 (PMC11269152; doi:10.3389/frph.2024.1304749)
Supplement: Supplementary file 1 [file Table1.docx]

**Supplemental material**

**Examining modification of the associations between air pollution and birth outcomes by neighborhood deprivation in a North Carolina birth cohort, 2011-2015**

Kristen Cowan, Alison K. Krajewski, Monica P. Jimenez, Thomas J. Luben, Lynne C. Messer, Kristen M. Rappazzo


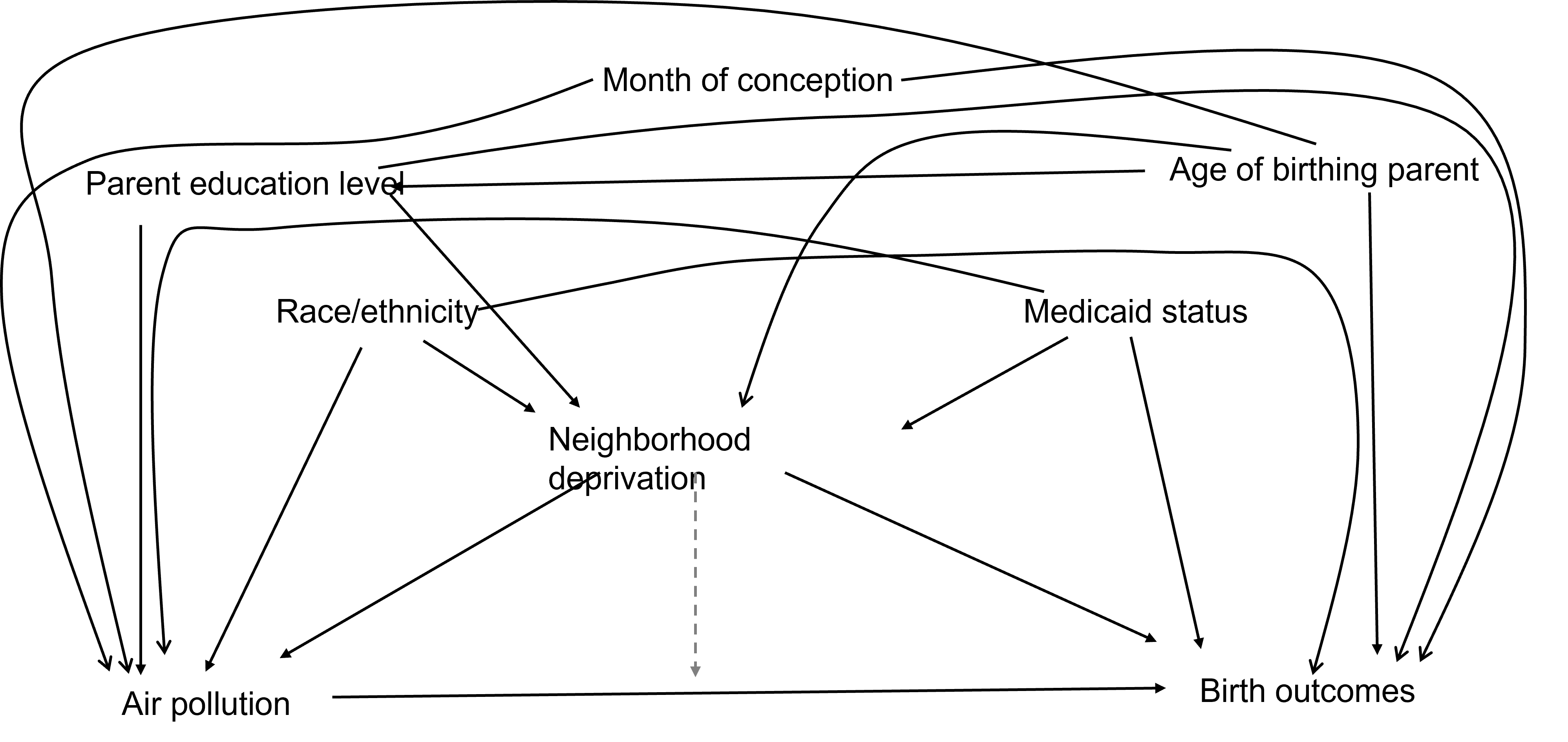


**Supplemental Figure S.1: Directed Acyclic Graph used to identify potential confounders, with dashed arrow indicating potential modifier of interest.**


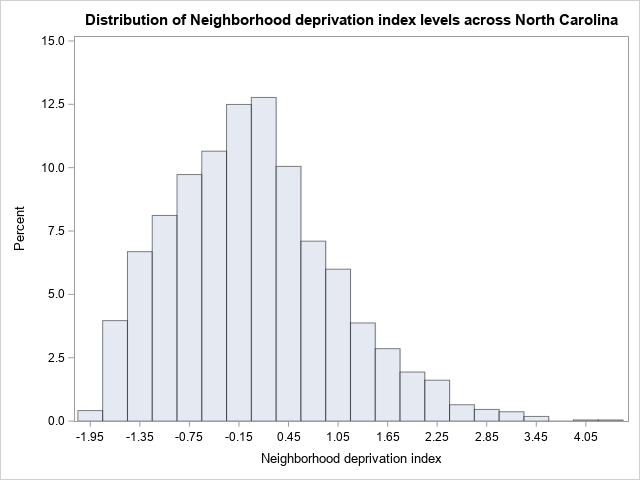


**Supplemental Figure S.2**: **Distribution of neighborhood deprivation index across North Carolina at census tract level**

**Supplemental Table S.1: Risk differences (95% CI) per 10,000 for air pollution exposure during pregnancy and preterm birth, stratified by neighborhood deprivation**

| **Pollutant by Exposure Window** | **All areas** | **Low NDI** | **Medium NDI** | **High NDI** |
| --- | --- | --- | --- | --- |
| PM_2.5_ (µg/m^3^)^1^ |  |  |  |  |
| Entire pregnancy | -23.56 (-30.83, -16.29) | -23.79 (-35.04, -12.54) | -18.51 (-29.39, -7.63) | 0.43 (-20.61, 21.47) |
| Trimester 1 | -16.23 (-21.69, -10.76) | -11.33 (-19.66, -2.99) | -15.96 (-24.22, -7.70) | -9.54 (-25.12, 6.04) |
| Trimester 2 | -23.60 (-29.06, -18.15) | -23.54 (-32.25, -14.83) | -20.59 (-28.56, -12.62) | -13.19 (-29.26, 2.88) |
| O_3_ (ppb)^2^ |  |  |  |  |
| Entire pregnancy | 8.52 (-2.32, 19.37) | 3.68 (-13.02, 20.39) | 6.60 (-9.68, 22.89) | 12.83 (-16.73, 42.40) |
| Trimester 1 | -0.24 (-8.09, 7.62) | 9.86 (-2.02, 21.74) | -8.13 (-20.06, 3.81) | -16.39 (-38.38, 5.60) |
| Trimester 2 | -9.32 (-17.44, -1.19) | -8.57 (-21.10, 3.96) | -11.85 (-24.08, 0.38) | -16.21 (-38.66, 6.24) |
| NO_2_ (ppb)^3^ |  |  |  |  |
| Entire pregnancy | -39.53 (-50.77, -28.28) | -42.78 (-60.80, -24.76) | -44.24 (-61.79, -26.68) | 34.70 (4.84, 64.56) |
| Trimester 1 | -42.44 (-53.10, -31.77) | -46.84 (-63.88, -29.80) | -46.83 (-63.45, -30.21) | 29.00 (0.52, 57.47) |
| Trimester 2 | -45.30 (-56.14, -34.46) | -47.40 (-64.69, -30.10) | -49.01 (-65.93, -32.09) | 17.87 (-11.17, 46.92) |

1: Risk differences are per 1 µg/m3 increase in PM_2.5_

2: Risk differences are per 4 ppb increase in O_3_

3: Risk differences are per 7 ppb increase in NO_2_

All models were adjusted for birthing parent race/ethnicity (white, non-Hispanic as reference), birthing parent age at delivery (centered at age 26 with a quadratic term), marital status (married as reference), Medicaid status (no as reference), education (>HS as reference), and month of conception (index variable as referent)

**Supplemental Table S.2: Prevalence differences (95% CI) per 10,000 births for air pollution exposure during pregnancy and birth defects, stratified by neighborhood deprivation**

| **Pollutant by Exposure Window** | **All areas** | **Low NDI** | **Medium NDI** | **High NDI** |
| --- | --- | --- | --- | --- |
| **PM_2.5_ (µg/m^3^)^1^** |  |  |  |  |
| Any Birth Defect (N=17,652) | -10.58 (-14.01, -7.15) | -12.58 (-17.18, -7.97) | -1.04 (-5.66, 3.59) | -6.48 (-14.01, 1.05) |
| Pulmonary Valve atresia/stenosis (N=478) | 0.34 (-0.23, 0.92) | 0.95 (0.20, 1.69) | 0.04 (-0.77, .86) | 5.30 (-16.47, 27.07) |
| Tetralogy of Fallot (TOF) (N=245) | -0.49 (-10.59, 9.60) | -6.22 (-23.36, 10.92) | 2.13 (-12.45, 16.70) | 7.67 (-22.84, 38.18) |
| Atrioventricular septal defects (N=242) | 0.99 (-0.33, 0.52) | -0.70 (-17.10, 15.70) | 0.40 (-0.10, 0.90) | -0.30 (-25.87, 25.26) |
| Limb reduction defects (N=80) | -2.31 (-15.65, 12.23) | 1.40 (-17.40, 20.30) | -2.97 (-19.75, 15.69) | 6.79 (-23.51, 37.09) |
| Gastroschisis (N=213) | -2.23 (-16.69, 12.23) | -5.30 (-24.40, 13.80) | 0.87 (-13.95, 15.69) | -5.14 (-34.15, 23.87) |
| **O_3_ (ppb)^2^** |  |  |  |  |
| Any Birth Defect (N=17,652) | -8.99 (-13.96, -4.03) | -4.50 (-8.60, -0.40) | -2.35 (-6.43, 1.72) | -4.61 (-10.79, 1.57) |
| Pulmonary Valve atresia/stenosis (N=478) | -0.34 (-1.19, .50) | 0.28 (-0.29, .85) | -0.59 (-1.27, 0.09) | 0.07 (-16.43, 16.57) |
| Tetralogy of Fallot (TOF) (N=245) | -2.80 (-18.70, 13.09) | -4.67 (-18.92, 9.60) | 2.23 (-9.97, 14.42) | 3.63 (-18.06, 25.32) |
| Atrioventricular septal defects (N=242) | 0.08 (-0.55, 0.72) | 1.07 (-12.03, 14.17) | 0.35 (-0.13, 0.83) | 4.97 (-16.46, 26.39) |
| Limb reduction defects (N=80) | -5.01 (-23.67, 13.65) | -2.84 (-19.08, 13.40) | 1.21 (-14.31, 16.72) | 1.38 (-18.77, 21.52) |
| Gastroschisis (N=213) | -1.95 (-22.96, 19.07) | -0.22 (-15.43, 14.99) | -3.48 (-16.38, 9.42) | -4.76 (-28.15, 18.63) |
| **NO_2_ (ppb)^3^** |  |  |  |  |
| Any Birth Defect (N=17,652) | -45.09 (-51.88, -38.30) | -37.65 (-47.34, -27.96) | -22.70 (-31.94, -13.47) | -32.31 (-46.10, -18.53) |
| Pulmonary Valve atresia/stenosis (N=478) | -15.58 (-28.08, -3.08) | -0.90 (-2.42, .62) | -1.47 (-2.73, -.22) | -5.20 (-41.33, 30.93) |
| Tetralogy of Fallot (TOF) (N=245) | -11.72 (-33.80, 10.36) | -16.46 (-51.16, 18.25) | -11.10 (-41.87, 19.67) | -5.22 (-47.44, 37.00) |
| Atrioventricular septal defects (N=242) | -12.31 (-23.93, -0.70) | -10.33 (-44.66, 23.99) | -11.07 (-22.49, 0.34) | -9.26 (-55.98, 37.75) |
| Limb reduction defects (N=80) | -10.02 (-62.42, 42.38) | -5.69 (-47.47, 36.08) | -10.49 (-46.27, 25.30) | -19.71 (-80.55, 41.13) |
| Gastroschisis (N=213) | -7.10 (-35.50, 21.30) | -13.77 (-51.02, 23.48) | -9.51 (-40.37, 21.35) | 2.87 (-42.12, 47.87) |

1: Prevalence differences are per 1 µg/m3 increase in PM_2.5_

2: Prevalence differences are per 4 ppb increase in O_3_

3: Prevalence differences are per 7 ppb increase in NO_2_

All models were adjusted for birthing parent race/ethnicity (white, non-Hispanic as reference), birthing parent age at delivery (centered at age 26 with a quadratic term), education (>HS as reference), and month of conception (index variable as referent)

**Supplemental Table S.3: Prevalence differences (95% CI) per 10,000 births across trimester 1 for air pollution exposure during pregnancy and birth defects additionally adjusted models, stratified by neighborhood deprivation**

| **Pollutant by Exposure Window** | **All areas** | **Low NDI** | **Medium NDI** | **High NDI** |
| --- | --- | --- | --- | --- |
| **PM_2.5_ (µg/m^3^)^1^** | |  |  |  |
| Any Birth Defect (N=17,652) | -10.72 (-14.14, -7.30) | -17.12 (-22.48, -11.77) | -3.03 (-8.19, 2.13) | -10.53 (-19.42, -1.64) |
| Pulmonary Valve atresia/stenosis (N=478) | 0.33 (-0.24, 0.90) | 5.41 (-1.55, 12.38) | 0.13 (-0.76, 1.02) | 3.83 (-23.00, 30.66) |
| Tetralogy of Fallot (TOF) (N=245) | -0.70 (-10.59, 9.20) | -6.18 (-23.36, 11.00) | -0.78 (-19.17, 17.61) | 6.67 (-32.99, 46.33) |
| Atrioventricular septal defects (N=242) | 0.10 (-0.33, 0.52) | -2.37 (-24.31, 19.57) | 4.41 (-8.34, 17.15) | -0.22 (-36.50, 36.05) |
| Limb reduction defects (N=80) | -2.21 (-15.63, 11.21) | 0.81 (-23.78, 25.39) | -5.82 (-27.41, 15.77) | 2.70 (-39.75, 45.15) |
| Gastroschisis (N=213) | -3.26 (-16.23, 9.72) | -2.61 (-28.25, 23.04) | -1.07 (-20.70, 18.56) | -5.29 (-43.73, 33.15) |
| **O_3_ (ppb)^2^** |  |  |  |  |
| Any Birth Defect (N=17,652) | -9.30 (-14.26, -4.35) | -12.12 (-19.97, -4.27) | -6.73 (-14.18, 0.72) | -12.95 (-25.56, -0.35) |
| Pulmonary Valve atresia/stenosis (N=478) | -0.32 (-1.16, 0.53) | 0.50 (-0.89, 1.90) | -10.92 (-21.75, -0.08) | 1.16 (-37.24, 39.56) |
| Tetralogy of Fallot (TOF) (N=245) | -2.80 (-18.70, 13.09) | -10.21 (-40.69,20.27) | 0.75 (-22.77, 24.27) | 5.40 (-49.48, 60.29) |
| Atrioventricular septal defects (N=242) | 0.08 (-0.55, 0.71) | 4.57 (-25.98, 35.12) | -0.96 (-18.20, 16.27) | 1.42 (-43.60, 46.44) |
| Limb reduction defects (N=80) | -5.01 (-23.67, 13.65) | 1.06 (-35.89, 38.01) | -11.20 (-41.91, 19.50) | 4.94 (-52.97, 62.84) |
| Gastroschisis (N=213) | -1.95 (-22.96, 19.07) | -2.32 (-41.78, 37.14) | -2.55 (-30.86, 25.76) | -3.45 (-55.97, 49.08) |
| **NO_2_ (ppb)^3^** | |  |  |  |
| Any Birth Defect (N=17,652) | -44.67 (-51.45, -37.89) | -56.02 (-67.47, -44.57) | -30.23 (-40.70, -19.76) | -45.04 (-61.50, -28.58) |
| Pulmonary Valve atresia/stenosis (N=478) | -15.96 (28.83, -3.09) | -0.45 (-2.23, 1.33) | -19.28 (-40.16, 1.59) | -9.63 (-62.10, 42.84) |
| Tetralogy of Fallot (TOF) (N=245) | -11.56 (-33.85, 10.74) | -17.74 (-60.50, 25.01) | -8.92 (-46.43, 28.78) | -8.65 (-67.37, 50.08) |
| Atrioventricular septal defects (N=242) | -12.45 (-24.24, -0.65) | -12.53 (-57.31, 32.24) | -13.45 (-40.68, 13.79) | -4.19 (-69.50, 61.12) |
| Limb reduction defects (N=80) | -10.02 (-62.43, 42.40) | -10.02 (-62.43, 42.40) | -7.58 (-55.62. 40.45) | -18.59 (-95.10, 57.92) |
| Gastroschisis (N=213) | -10.35 (-34.99, 14.29) | -6.28 (-59.10, 46.54) | -7.92 (-49.95, 34.11) | -4.78 (-71.34, 61.78) |

1: Prevalence differences are per 1 µg/m3 increase in PM_2.5_

2: Prevalence differences are per 4 ppb increase in O_3_

3: Prevalence differences are per 7 ppb increase in NO_2_

All models were adjusted for birthing parent race/ethnicity (white, non-Hispanic as reference), birthing parent age at delivery (centered at age 26 with a quadratic term), education (>HS as reference), and month of conception (index variable as referent)

**Supplemental Table S.4: Prevalence differences (95% CI) per 10,000 births for air pollution exposure during pregnancy and birth defects for specific weeks (gestational weeks 3-8), stratified by neighborhood deprivation**

| **Pollutant by Exposure Window** | **All areas** | **Low NDI** | **Medium NDI** | **High NDI** |
| --- | --- | --- | --- | --- |
| **PM_2.5_ (µg/m^3^)^1^** |  |  |  |  |
| Pulmonary valve atresia or stenosis | 0.32 (-0.11, 0.75) | 0.58 (-0.04, 1.20) | -0.11 (-0.11, -0.79) | 4.79 (-13.68, 23.26) |
| Tetralogy of Fallot | -1.21 (-9.55, 7.13) | -5.06 (-19.57, 9.45) | 0.24 (-12.10, 12.58) | 3.70 (-20.48, 27.88) |
| Atrioventricular septal defects | 0.10 (-0.22, 0.41) | -0.10 (-13.70, 13.49) | 0.26 (-0.23, 0.74) | -0.62 (-23.70, 22.46) |
| Limb reduction defects | 0.89 (-7.86, 9.64) | 1.67 (-14.18, 17.52) | -2.34 (-17.25, 12.57) | 5.35 (-20.11, 30.82) |
| **O_3_ (ppb)^2^** |  |  |  |  |
| Pulmonary valve atresia or stenosis | -0.03 (-0.40, 0.35) | 0.32 (-0.18, 0.83) | -0.49 (-1.11, 0.23) | 0.01 (-15.30, 15.33) |
| Tetralogy of Fallot | -0.36 (-7.71, 6.99) | -4.09 (-17.11, 8.93) | 2.06 (-9.56, 13.67) | 0.80 (-19.16, 20.75) |
| Atrioventricular septal defects | 0.24 (-0.04, 0.52) | 1.30 (-10.42, 13.01) | 0.31 (-0.13, 0.75) | 4.28 (-15.87, 24.43) |
| Limb reduction defects | -0.44 (-8.02, 7.14) | -1.86 (-15.50, 11.77) | -1.71 (-16.95, 13.53) | 3.99 (-14.88, 22.86) |
| **NO_2_ (ppb)^3^** |  |  |  |  |
| Pulmonary valve atresia or stenosis | -1.03 (-1.83, -0.22) | -1.02 (-2.40, 0.36) | -1.36 (-2.58, -0.14) | -2.46 (-37.82, 32.89) |
| Tetralogy of Fallot | -10.58 (-28.99, 7.83) | -14.00 (-47.59, 19.59) | -13.32 (-44.47, 17.83) | -2.94 (-43.01, 37.13) |
| Atrioventricular septal defects | -13.28 (-23.32, -3.24) | -9.41 (-42.26, 23.44) | -15.74 (-32.66, 1.17) | -11.14 (-57.77, 35.48) |
| Limb reduction defects | -7.89 (-29.66, 13.89) | -3.53 (-41.63, 34.57) | -12.04 (-50.26, 26.17) | -19.59 (-81.05, 41.86) |

1: Prevalence differences are per 1 µg/m3 increase in PM_2.5_

2: Prevalence differences are per 4 ppb increase in O_3_

3: Prevalence differences are per 7 ppb increase in NO_2_

All models were adjusted for birthing parent race/ethnicity (white, non-Hispanic as reference), birthing parent age at delivery (centered at age 26 with a quadratic term), education (>HS as reference), and month of conception (index variable as referent)

**Supplemental Table S.5: Risk differences (95% CI) per 10,000 births for air pollution exposure during pregnancy and interaction with neighborhood deprivation for preterm birth**

| **Pollutant** | **Risk Difference for** | **Entire Pregnancy** | **Trimester 1** | **Trimester 2** |
| --- | --- | --- | --- | --- |
| **PM_2.5_ (µg/m^3^)^1^** | PM_2.5_ | -19.80 (-30.54, -9.07) | -8.83 (-16.33, -1.33) | -20.11 (-27.98, -12.24) |
|  | Medium NDI | 77.81 (-63.46, 219.09) | 138.04 (41.22, 234.85) | 78.40 (-19.93, 176.72) |
|  | High NDI | -75.56 (-291.68, 140.55) | 93.91 (-51.62, 239.43) | 55.59 (-97.12, 208.30) |
|  | PM_2.5_*Medium NDI | 57.85 (-76.63, 192.33) | 122.95 (30.79, 215.10) | 58.07 (-35.90, 152.05) |
|  | PM_2.5_*High NDI | -76.55 (-275.68, 122.58) | 86.26 (-48.12, 220.64) | 40.68 (-100.22, 181.59) |
| **O_3_ (ppb)^2^** | O_3_ | 8.29 (-5.74, 22.33) | 4.60 (-4.07, 13.27) | -6.74 (-15.77, 2.30) |
|  | Medium NDI | 84.20 (-89.63, 258.04) | 165.29 (75.41, 255.18) | 108.90 (15.44, 202.35) |
|  | High NDI | 71.30 (-172.43, 315.04) | 173.03 (47.11, 298.95) | 137.09 (7.94, 266.23) |
|  | O_3_*Medium NDI | 86.20 (-85.46, 257.87) | 164.37 (75.67, 253.07) | 106.54 (14.30, 198.78) |
|  | O_3_*High NDI | 74.23 (-165.02, 313.49) | 172.52 (48.88, 296.16) | 134.64 (7.78, 261.50) |
| **NO_2_ (ppb)^3^** | NO_2_ | -41.95 (-58.74, -25.15) | -42.96 (-58.00, -27.93) | -43.11 (-58.17, -28.06) |
|  | Medium NDI | 69.88 (22.90, 116.87) | 66.07 (24.23, 107.92) | 70.89 (29.43, 112.34) |
|  | High NDI | -29.40 (-100.74, 41.94) | -1.43 (-65.00, 62.13) | 8.59 (-54.33, 71.52) |
|  | NO_2_*Medium NDI | 63.89 (18.26, 109.53) | 60.18 (19.47, 100.90) | 64.62 (24.33, 104.91) |
|  | NO_2_*High NDI | -26.26 (-94.50, 41.97) | -.37 (-61.30, 60.55) | 9.08 (-51.19, 69.35) |

1: Risk differences are per 1 µg/m3 increase in PM_2.5_

2: Risk differences are per 4 ppb increase in O_3_

3: Risk differences are per 7 ppb increase in NO_2_

All models were adjusted for birthing parent race/ethnicity (white, non-Hispanic as reference), birthing parent age at delivery (centered at age 26 with a quadratic term), marital status (married as reference), Medicaid status (no as reference), education (>HS as reference), and month of conception (index variable as referent)

**Supplemental Table S.6: Prevalence differences (95% CI) per 10,000 births for air pollution exposure during pregnancy and interaction with neighborhood deprivation for birth defects**

| **Pollutant** | **Prevalence Difference for** | **Any Defect** | **Pulmonary valve atresia or stenosis** | **Tetralogy of fallot** |
| --- | --- | --- | --- | --- |
| **PM_2.5_ (µg/m^3^)^1^** | PM_2.5_ | -12.58 (-17.10, -8.07) | 0.82 (0.11, 1.53) | -6.31 (-22.63, 10.01) |
|  | Medium NDI | -66.08 (-128.78, -3.38) | 10.09 (-0.13, 20.32) | -69.29 (-281.47, 142.89) |
|  | High NDI | -25.90 (-112.28, 60.48) | 1.88 (-14.07, 17.83) | -110.97 (-440.82, 218.87) |
|  | PM_2.5_*Medium NDI | -67.96 (-127.59, -8.32) | 10.12 (0.50, 19.75) | -67.74 (-270.41, 134.93) |
|  | PM_2.5_*High NDI | -32.61 (-112.78, 47.57) | 2.91 (-11.71, 17.53) | -104.50 (-409.01, 200.01) |
| **O_3_ (ppb)^2^** | O_3_ | -4.21 (-8.26, -0.16) | 0.29 (-0.25, 0.82) | -4.74 (-18.88, 9.41) |
|  | Medium NDI | 16.49 (-42.80, 75.79) | 11.21 (2.25, 20.18) | -64.93 (-258.23, 128.36) |
|  | High NDI | 36.66 (-39.79, 113.11) | 6.47 (-6.04, 18.98) | -72.02 (-331.47, 187.41) |
|  | O_3_*Medium NDI | 14.43 (-42.10, 70.96) | 10.65 (2.20, 19.10) | -62.66 (-247.92, 122.60) |
|  | O_3_*High NDI | 31.93 (-39.54, 103.40) | 6.51 (-5.05, 18.08) | -68.36 (-310.94, 174.23) |
| **NO_2_ (ppb)^3^** | NO_2_ | -38.74 (-18.25, -29.22) | -0.99 (-2.45, 0.48) | -15.01 (-49.43, 19.42) |
|  | Medium NDI | 3.20 (-25.09, 31.49) | 3.30 (-0.99, 7.59) | -4.01 (-99.70, 91.67) |
|  | High NDI | 25.48 (-14.02, 65.15) | 3.80 (-2.86, 10.47) | -15.84 (-97.36, 65.69) |
|  | NO_2_*Medium NDI | -24.85 (-45.92, 2.23) | 1.75 (-1.98, 5.47) | -4.07 (-128.30, 120.17) |
|  | NO_2_*High NDI | -8.49 (-38.89, 21.92) | 2.97 (-2.03, 7.97) | -10.55 (-108.34, 87.23) |

| **Pollutant** | **Prevalence Difference for** | **Atrioventricular septal defects** | **Limb reduction defects** | **Gastroschisis** |
| --- | --- | --- | --- | --- |
| **PM_2.5_ (µg/m^3^)^1^** | PM_2.5_ | -9.60 (-4.92, -4.27) | 1.73 (-16.30, 19.75) | -4.33 (-23.14, 14.49) |
|  | Medium NDI | -39.10 (-114.15, 35.96) | 47.33 (-191.39, 286.04) | -42.45 (-275.71, 190.81) |
|  | High NDI | -43.37 (-148.54, 61.80) | -26.49 (-365.45, 312.47) | 21.49 (-328.09, 371.06) |
|  | PM_2.5_*Medium NDI | -42.33 (-113.61, 28.95) | 44.33 (-183.57, 272.23) | -40.72 (-264.83, 183.39) |
|  | PM_2.5_*High NDI | -47.42 (-144.82, 49.98) | -19.97 (-334.79, 294.85) | 16.11 (-308.22, 340.43) |
| **O_3_ (ppb)^2^** | O_3_ | -0.03 (-1.66, 1.60) | -3.71 (-18.87, 11.45) | 1.14 (13.77, 16.05) |
|  | Medium NDI | 10.17 (-14.13, 34.46) | -44.49 (-273.49, 184.51) | 46.36 (-155.79, 248.50) |
|  | High NDI | 27.10 (-3.95, 58.15) | -23.42 (-281.24, 234.40) | 75.25 (-183.90, 334.41) |
|  | O_3_*Medium NDI | 10.15 (-12.95, 33.25) | -43.52 (-260.82, 173.78) | 44.66 (-149.49, 238.80) |
|  | O_3_*High NDI | 24.80 (-4.21, 53.82) | -22.71 (-265.28, 219.90) | 70.27 (-172.81, 313.35) |
| **NO_2_ (ppb)^3^** | NO_2_ | -6.24 (-9.95, -2.54) | -3.61 (-43.08, 35.87) | -11.42 (-48.64, 25.80) |
|  | Medium NDI | 5.50 (-5.67, 16.66) | 15.52 (-93.25, 124.48) | 6.81 (-91.84, 105.46) |
|  | High NDI | -13.66 (-29.23, 1.92) | 38.45 (-112.61, 189.51) | -16.08 (-155.82, 123.66) |
|  | NO_2_*Medium NDI | 0.99 (-8.49, 10.47) | 3.76 (-89.76, 97.28) | -1.26 (-87.02, 84.51) |
|  | NO_2_*High NDI | -11.55 (-23.40, 0.30) | 24.23 (-91.62, 140.07) | -14.34 (-121.78, 93.11) |

1: Prevalence differences are per 1 µg/m3 increase in PM_2.5_

2: Prevalence differences are per 4 ppb increase in O_3_

3: Prevalence differences are per 7 ppb increase in NO_2_

All models were adjusted for birthing parent race/ethnicity (white, non-Hispanic as reference), birthing parent age at delivery (centered at age 26 with a quadratic term), education (>HS as reference), and month of conception (index variable as referent)
